# Androgen receptor-mediated pharmacogenomic expression quantitative trait loci: implications for breast cancer response to AR-targeting therapy

**DOI:** 10.1186/s13058-024-01861-2

**Published:** 2024-07-04

**Authors:** Huanyao Gao, Lixuan Wei, Shreya Indulkar, Thanh Thanh. L. Nguyen, Duan Liu, Ming-Fen Ho, Cheng Zhang, Hu Li, Richard M. Weinshilboum, James N. Ingle, Liewei Wang

**Affiliations:** 1 Department of Molecular Pharmacology and Experimental Therapeutics, Mayo Clinic, 200 First Street Southwest, Rochester, MN 55905 USA; 2 Department of Oncology, Mayo Clinic, Rochester, MN USA

**Keywords:** PGx-eQTL, Breast cancer, Endocrine therapy, Aromatase inhibitor, GWAS, Pharmacogenomics, Androgen receptor

## Abstract

**Background:**

Endocrine therapy is the most important treatment modality of breast cancer patients whose tumors express the estrogen receptor α (ERα). The androgen receptor (AR) is also expressed in the vast majority (80–90%) of ERα-positive tumors. AR-targeting drugs are not used in clinical practice, but have been evaluated in multiple trials and preclinical studies.

**Methods:**

We performed a genome-wide study to identify hormone/drug-induced single nucleotide polymorphism (SNP) genotype - dependent gene-expression, known as PGx-eQTL, mediated by either an AR agonist (dihydrotestosterone) or a partial antagonist (enzalutamide), utilizing a previously well characterized lymphoblastic cell line panel. The association of the identified SNPs-gene pairs with breast cancer phenotypes were then examined using three genome-wide association (GWAS) studies that we have published and other studies from the GWAS catalog.

**Results:**

We identified 13 DHT-mediated PGx-eQTL loci and 23 Enz-mediated PGx-eQTL loci that were associated with breast cancer outcomes post ER antagonist or aromatase inhibitors (AI) treatment, or with pharmacodynamic (PD) effects of AIs. An additional 30 loci were found to be associated with cancer risk and sex-hormone binding globulin levels. The top loci involved the genes IDH2 and TMEM9, the expression of which were suppressed by DHT in a PGx-eQTL SNP genotype-dependent manner. Both of these genes were overexpressed in breast cancer and were associated with a poorer prognosis. Therefore, suppression of these genes by AR agonists may benefit patients with minor allele genotypes for these SNPs.

**Conclusions:**

We identified AR-related PGx-eQTL SNP-gene pairs that were associated with risks, outcomes and PD effects of endocrine therapy that may provide potential biomarkers for individualized treatment of breast cancer.

**Supplementary Information:**

The online version contains supplementary material available at 10.1186/s13058-024-01861-2.

## Background

Endocrine therapy is the most important therapy for the majority of breast cancer (BC) patients (approximately 70%) whose tumors express estrogen receptor alpha (ERα). Endocrine therapies, including ER antagonists (selective ER modulators [SERMs], selected ER degraders [SERDs]) and estrogen biosynthesis inhibitors (aromatase inhibitor [AI]), have been widely used clinically in adjuvant, neo-adjuvant, and preventative settings. In order to better understand individually variable clinical responses, efficacy, and adverse effects, we performed genome-wide association studies (GWAS) and identified single-nucleotide polymorphisms (SNPs) associated with multiple endocrine therapy treatment response phenotypes including disease-free survival in patients treated with AIs [1], the development of breast cancer in women treated with tamoxifen when used for prevention [2], musculoskeletal adverse events in women treated with AIs as adjuvant therapy [3], fragility bone fractures in women treated with AIs [4], and suppression of estrone (E1) and estradiol (E2) plasma levels in women treated with the AI anastrozole as adjuvant therapy [5]. These GWAS have identified SNPs which are expression quantitative trait loci (eQTLs) only after exposure to ERα ligands and contribute to individual variations in response to endocrine therapy [1–5].

The androgen receptor (AR) is also expressed in the vast majority (80–90%) of ER positive (ER+) BC [6, 7]. Even in triple negative breast cancer (TNBC), up to 40% of cases express AR [8]. However, unlike ERα, which promotes BC tumor growth, the functions and roles of AR and AR modulators in BC are controversial. Some studies reported that AR promotes BC tumor growth and metastasis while others reported that androgens primarily exhibit tumor suppressive effects [6, 7, 9]. Moreover, since androgens are substrates of aromatase in the biosynthesis of estrogens, it is not surprising that ER- or aromatase-targeting endocrine therapies have been reported to modulate the relative levels of androgens, and to activate AR-downstream signaling [10, 11]. Natural AR ligands showed limited effects in treating ER + BC in trials dating back to the 1950s [12, 13], while a trial of AR antagonist in combination with an aromatase inhibitor showed improved prognosis in a subpopulation of BC patients with high AR and low ERα levels [14]. Recently, we reported that the benefit of RAD-140 (an AR agonist) and enzalutamide (Enz, a partial AR antagonist) in breast cancer cell lines was dependent on the ratio of AR to ER level. Specifically, Enz was more efficacious in ERα-positive disease with a low AR/ER ratio whereas the AR agonist was more efficacious in patients with a high AR/ER ratio [15]. Although the benefit of AR agonist/antagonist in BC are still of much debate, it is certain that AR-regulated gene transcription plays a role in BC pathophysiology.

Both AR and ER are ligand-activating nuclear receptors which, once activated, can bind to specific DNA sequence motifs and regulate gene transcription. Genetic variants or SNPs which affect DNA binding of those ligand-activated nuclear receptors often result in differing gene expression. Those genetic variants or SNPs are expression quantitative trait loci (eQTL) only after exposure to ligands. These eQTLs that are associated with certain ligands or drugs are referred to as pharmacogenomic eQTLs (PGx-eQTL), whereas those that are not associated with pharmacological treatments are referred as baseline eQTLs (Additional file 1: Fig. S1**A**). Previously, both our group [2, 16] and others [17, 18] have identified a number of SNP-gene pairs of PGx-eQTL signals that are implicated in drug-response in breast cancer and psychiatric disorders. Furthermore, using the glucocorticoid receptor (GR) as a model TF, we have reported the genome-wide identification of PGx-eQTLs using a lymphoblastoid cell line (LCL) panel, and demonstrated the clinical relevance of these PGx-eQTLs for clinically relevant phenotypes via overlapping with GWAS data [19]. In this manuscript, we have applied the same strategy to interrogate the AR to identify PGx-eQTL SNP-gene pairs induced by either androgens (dihydrotestosterone [DHT]) or Enz, which have been reported to induce reprogramming of chromatin and to induce an alternative cistrome as compared to an androgen-inducible cistrome [20–22], to integrate with our previous BC GWAS data with the goal of identifying silent non-coding genetic risk variants important in breast cancer.

## Methods

### Experimental design for androgen receptor modulator-induced PGx eQTLs

In order to study the genotype-related AR-mediated PGx-eQTLs, we employed a panel of 30 human LCLs, subjected to treatments with vehicle, AR agonist (DHT), AR partial antagonist (Enz), or dual-treatments with both DHT and Enz (Additional file 1: Fig S1b). The LCL panel has been previously well characterized by genotyping and expression microarray [23] and have been successfully used in a range of pharmacogenomic studies [19, 23, 24], and thus serve as a potential model system for our purpose. Since AR is expressed at relatively low levels in LCLs, to ensure the signals were AR-specific, we carefully selected 30 LCLs so that 15 of the cell lines expressed variable amounts of AR, while the other 15 cell lines expressed essentially no AR. Followed the RNA sequencing of LCLs with different treatments, we performed PGx-eQTL analysis using gene expression fold change between drug-treated and vehicle-treated for each individual cell, which was demonstrated to be a successful strategy previously for PGx-eQTL study of GR in order to minimize baseline expression differences among cells for PGx-eQTL analysis [19]. To ensure the PGx-eQTL signals are indeed AR-targeting ligand/drugs-related, we took three more steps to filter the signals. (1) Any signals that were repeated in AR-null samples were considered to be AR-independent and were excluded from the signals from AR-expressed samples; (2) Any signals in the presence of a drug that were not reversible by dual-treatment were considered treatment independent or ambiguous and were excluded from the drug-specific signals; (3) All signals should locate in genomic regions containing at least one AR-binding site that has been previously identified via AR ChIPseq analysis performed in prostate or breast tissues. The last filter step also helped to confirm the relevance of PGx-eQTLs identified in the LCL model system in those androgen-responsive tissues such as breast and prostate.

Finally, clinical relevance of the AR mediated PGx-eQTL signals were identified by overlapping the PGx-eQTL SNP with previously published GWAS results [1, 2, 5, 25].

### Cell culture and drug conditions

LCLs were obtained from the Coriell Institute. LCLs were cultured in RPMI 1640 supplemented with 15% FBS and 1% penicillin/streptomycin, under 37 °C and 5% CO_2_. Prior to DHT/Enz treatment, all cells were grown in 5% charcoal-stripped FBS supplemented media for 24 h, then either DHT in Ethanol (final concentration 10nM) or Enz in DMSO (final concentration 100nM), combination of both drugs, or pure vehicle of 0.1%DMSO/0.1%Ethanol was added to the culture and incubated for 8 h before harvesting by centrifugation and snap frozen in trizol lysis buffer.

### RNA-sequencing experiments

Total RNA was extracted with the RNeasy RNA extraction kit per manufacturer’s instruction (Qiagen). RNA integrity was examined by Agilent Bioanalyzer. RNA-seq libraries were prepared with the TruSeq2 RNA Library Prep Kit v2 (Illumina). Pair-end 100 bp sequencing was performed on Illumina HiSeq 4000 at a sequencing depth of ∼25 million paired-end reads per sample. Raw RNA sequencing reads were aligned to the human genome GRCh37 (hg19) using STAR [26], and gene counts were performed using HTSEQ [27]. 8571 genes with raw counts less than 32 in at least 15 cell lines in at least one drug conditions were excluded from PGx-eQTL analysis. A total of 13,741 genes were included in PGx-eQTL analysis. Expression was normalized to library sizes by EdgeR package [28], and was further converted to log2 fold-changes over vehicle treatment for each cell line, respectively. Since AR is generally low expressed, we define cells with AR read counts less than 5 in all drug condition as AR-null, otherwise considered as AR expressed. A total of 15 cells were considered AR expressed and 15 cells were considered as AR-null. Traditional differential expression analysis was performed using EdgeR package [28].

### LCL genotype data quality control

LCLs from unrelated individuals were previously genotyped with Illumina HumanHap550K and HumanExon510S-Duo Bead Chips in our laboratory and deposited in Gene Expression Omnibus (GEO accession: GSE23120) [23]. The relatedness (identity-by-descent) has been analyzed by the plink -genome command. For all LCL panel data, no relatedness or ancestry has been found. Genotyping data was extracted with minor allele frequency of at least 0.2, maximum missing values of 5%, and Hardy-Weinberg test p-value > 0.001 using plink (www.cog-genomics.org/plink/1.9/) [29] for AR-expressed and AR-null cells, respectively.

### Pharmacogenomic (PGx) -eQTL analysis

PGx-eQTL analysis for gene expression as fold changes upon drug treatment over vehicle treatment was performed using R package MatrixEQTL with the ANOVA model [30]. The analysis focuses on cis-eQTL SNPs located within up to 200 kbp up- or downstream of the corresponding gene in AR-expressed and AR-null cell lines, respectively. Trans-eQTL, sex chromosome and mitochondrial genome were excluded from all analysis. P-values ≤ 0.005 was preliminarily considered as significant. AR-independent signals were identified from AR-null cells and excluded from AR-expressed cells. Treatment independent or ambiguous signals were identified from DHT-ENZ dual-treatment cells since DHT and ENZ were considered to antagonize each other in AR signalling and signals that remained in the dual-treatment (non-reversible) were excluded from final results. Finally, AR ChIPseq data were used to identify PGx-eQTL SNP sites having identified AR binding sites.

### AR ChIPseq data

Human AR ChIPseq peak data was downloaded from Remap2022 (https://remap2022.univ-amu.fr/) [31]. Sample types were examined to include only those experiments performed using breast and prostate normal or cancer tissues and cell lines. Downloaded data was mapped to hg38 reference genome and was converted to hg19 using LiftOver tools from UCSC genome browser website (https://genome.ucsc.edu/cgi-bin/hgLiftOver). Overlapping ChIPseq with PGx-eQTL SNP was performed using GenomicRanges package [32] on R.

### ChromHMM annotation of SNPs

ChromHMM annotation data was downloaded from HaploReg4.2 website (https://pubs.broadinstitute.org/mammals/haploreg/haploreg.php) [33], which hosted the multi-tissue ChromHMM 15-states and 25-states annotation of SNPs generated by Ernst and Kellis [34, 35]. ChromHMM 15-states were combined into 4 categories: TSS, including 1_TssA and 2_TssAFlnk, indicating transcription start sites; Transcr, including (3_TxFlnk, 4_Tx, and 5_TxWk), indicating transcription states; Enhancer, including 6_EnhG and 7_Enh, indicating enhancer status; and Repressed, including all other states, indicating heterochromatin, biovalent/poised TSS or enhancer or other inactive chromatin states. Since number of SNPs with breast tissue annotation was very limited, we summarized chromatin state annotation for each SNP across tissues and use the most prevalent annotation category as the states of the SNP. If two of the active states are equally prevalent, we presented as both, such as Enhancer_Mixed. If one active state and one repressed state was equally prevalent, we marked as Ambiguous.

### Homer motif analysis

Motif region was generated by expanding PGx-eQTL analysis significant SNPs location by 200 bp on both directions. For ChIPseq peak filtered motifs, only SNPs with AR ChIPseq overlapped were used to generate the motif regions. Motif analysis was then performed by findMotifsGenome.pl command of the homer software [36].

### SNPs overlapping with GWAS signals

GWAS of breast cancer prevention by SERMs (NSABP) (592 cases, 1171 controls, all Caucasian) [2], GWAS of breast cancer risk of relapse post-surgery and AI adjuvant therapy (MA27) (252 cases, 4406 controls, 95.7% Caucasian, 3.0% African, 1.3% Asian) [1], and GWAS of estradiol (E2) and estrone (E1) levels pre- and on- treatment with the AI anastrozole (M3) [5] (44 cases, 278 controls, all Caucasian) were previously published. All SNPs that were p-values ≤ 0.005 were extracted for matching with PGx-eQTL SNPs. GWAS catalogue data was downloaded from GWAS catalog website (https://www.ebi.ac.uk/gwas/) [25]. Due to relatively low SNP coverage of the LCL genotyping array, we also performed link disequilibrium (LD) block analysis by API query on LDlink database (https://ldlink.nih.gov/) via LDlinkR package on R [37]. r^2^ ≥ 0.8 between PGx-eQTL SNP and GWAS SNP were considered in the same LD block thus are likely to contribute to the same phenotype. The circos plots were generated using R package circlize [38]. Loci enrichment was performed using LOLA package in R software.

### Survival analysis

Survival analysis was performed using KMplotter(https://kmplot.com/analysis/) [39] multi-cohort breast cancer dataset using relapse free survival of all samples as phenotype and automatically selected cutoff for expression grouping.

### qRT-PCR validation experiments

Five additional AR-expressed LCL cell lines (CA10, CA12, CA22, CA85, and CA96) were cultured, DHT/Enz treated, and RNA extracted in the same fashion as described above. qRT-PCR was performed using the Power SYBR (Thermo Fisher). Gene expression analyses were performed using ΔΔCt method, and GAPDH was used as the internal reference. Three independent experiments were performed. The PGx-eQTL results as well as primer sequences of top signals in validation SNP-gene sets were summarized in Supplementary Table S1. To select the top signals to be validated, we followed the following criteria: (1) The signals are AR-expressed cells-specific, can be reversed by ENZ-DHT dual treatment, and have a nearby AR-binding site; (2) The signals have p-values < 0.0005 and |effect size| >0.5, or have p-values < 0.001 and |effect size| >0.4 and is a significant GWAS signals and; (3) Availability of at least one cell line among selected cell lines with a homozygous minor allele genotyped; (4) Expression can be detected by qRT-PCR (ΔCT < 30). After filtering, 15 signals were selected to be validated and 10 out of 15 signals were validated as presented in Supplementary Fig. S6.

## Results

### Landscape of AR modulator induced PGx – eQTLs

**Fig. 1:**
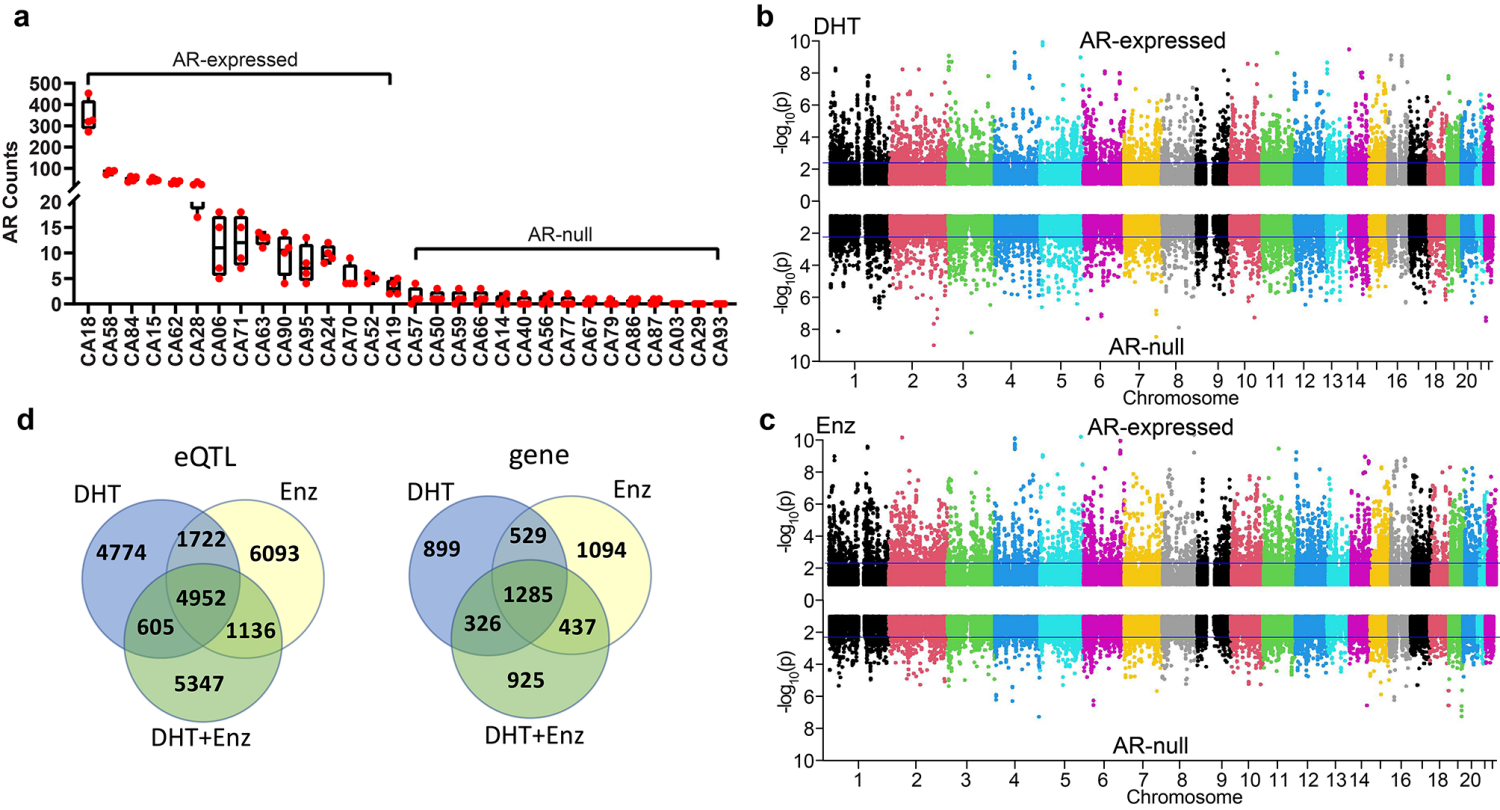
Landscape of AR PGx-eQTL signals. **a** AR expression in 30 LCLs based on RNAseq. Each box represents AR expression in four different treatment groups of a specific LCL as labeled at the bottom. **b** All DHT-induced PGx-eQTL signals in AR-expressed (upward) or AR-null cells (downward) are presented as Miami plots. X-axis represents the chromosomal location and the y-axis represents –log10(p-values) for PGx-eQTL analysis. The horizontal line indicated a tentative significance threshold. **c** All ENZ-induced PGx-eQTL signals in AR-expressed (upward) or AR-null cells (downward) are presented as a Miami plot. X-axis represents the chromosomal location and the y-axis represents –log10(p-values) for PGx-eQTL analysis. The horizontal line indicated a tentative significance threshold. **d** Venn diagrams comparing eQTL signals and relevant genes among DHT, ENZ, and DHT-ENZ double treatments

The expression of AR in 30 selected LCLs with 4 treatment conditions, including vehicles, was quantified by RNA-seq, and the AR expression level is presented in Fig. 1a. 15 LCLs had AR reads of 5 or more after at least one treatment and were considered “AR-expressed”, while the other 15 LCLs expressed minimal or no AR under any treatment condition and were considered AR-null. After MAF and Hardy-Weinberg test filter, overall AR-expressed and AR-null cells shared 83% of all SNPs and mostly consistent MAFs for corresponding SNPs (Additional file 1: Fig. S2). In order to test the AR-responsiveness of the AR-expressed cell lines, we first performed differential expression analysis by comparing signals between (a) DHT and vehicle treated and (b) Enz + DHT double treated and DHT only treated samples. The log2-fold changes for the two pairs of comparison were plotted against each other, and Enz has clearly reversed the expression changes induced by DHT (Additional file 1: Fig. S3a). We then examined expression of selected classic AR dependent genes such as FKBP5, PTGER4, and TMPRSS2 in LCLs and as expected, they were significantly induced by DHT, while reversed by Enz treatment (Additional file 1: Fig. S3b). The PGx-eQTL analysis was then performed separately for AR-expressed and AR-null cells, and the results from AR-null cells were used as negative controls for AR-independent gene transcription. The initial overall results of the landscape of PGx-eQTL analysis before further filtering are presented in Fig. 1b for DHT-induced signals and in Fig. 1c for Enz-induced signals as the Miami plots, with the top indicating signals from AR-expressing cells and the bottom indicating signals from AR-null cells. Quality and potential inflation of signals of the PGx-eQTL analysis were examined by quantile-quantile (q-q) plots (Additional file 1: Fig S4). A lambda value of less than 1.0 for all q-q plots was indicative of lack of inflation. Approximately 1% of signals were also identified in AR-null cells and were excluded from the overall results identified in AR-expressed cells.

DHT and Enz compete for AR-binding and thus antagonize each other in the activation of AR signalling, while used alone, they could each regulate the transcription of different subsets of genes [21, 22]. We therefore used signals from DHT/Enz dual-treatments to indicate the drug-specificity of DHT- or Enz-induced signals. At this point, we used a threshold of *p* ≤ 0.005 for the PGx-eQTL analysis partially due to the limitation of sample size. Using this cutoff, more than 40% the eQTLs (SNP-gene pairs) (5557 out of 12,053 for DHT and 6088 out of 13,953 for Enz) and approximately half of the eQTL genes (1611 out of 3039 for DHT and 1722 out of 3345 for Enz) were excluded since they were not reversible in the presence of the dual-treatments (Fig. 1d). Interestingly, approximately 25% of eQTL-SNP-gene pairs or 30% of eQTL genes were shared between DHT and Enz with the same directionalities but were not significant with Enz + DHT dual treatment.

**Fig. 2:**
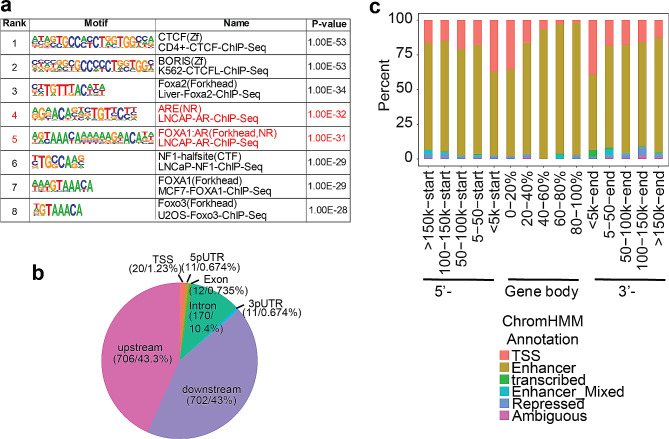
AR PGx-eQTL signals motif and cis-regulatory elements analysis. **a** Homer motif analysis of SNP periphery sequences from DHT/ENZ- induced PGx-eQTL signals, filtered by AR ChIPseq binding sites. Red text indicates classical AR binding motifs. **b** Piechart for SNPs locations relative to the corresponding PGx-eQTL genes, filtered by AR ChIPseq binding sites,. **c** Analysis of SNP-sites based ChromHMM cis-regulatory elements (RE) using all DHT/ENZ- induced PGx-eQTL signals, filtered by AR ChIPseq binding sites. X-axis indicates relative locations of SNPs to PGx-eQTL genes and color indicates cis-RE annotation by ChromHMM

One of the most common mechanisms for an eQTL is that the SNP might reside in the binding site of a TF which, in turn, regulates the expression of the target gene. Therefore, we required that the PGx-eQTL SNP sites be located within AR DNA binding sites identified by AR-ChIPseq performed in breast or prostate tissues, where AR is most highly expressed. After filtering, 749 (11.5%) of the SNPs from DHT- mediated PGx-eQTLs and 902 (11.5%) of the SNPs from Enz-mediated PGx-eQTL were found to reside within at least one AR ChIPseq peak. We then performed motif analysis using sequence motifs within 200 bp for PGx-eQTL SNPs after overlapping with AR ChIPseq, to further validate whether classic AR binding motifs were present within or near the genomic regions where PGx-eQTL SNPs were located. Indeed, AR-binding full sites were enriched with p-values of 1 × 10^− 32^ while FOXA1-AR co-binding sites had p-values of 1 × 10^− 31^ (Fig. 2a). The results were also consistent when we analyzed SNPs associated with DHT and Enz treatment separately (Additional file 1: Fig. S5).

Next, we proceeded to investigate the potential mechanisms of ways in which the SNPs might regulate gene expression. We first examined the genomic locations of the PGx-eQTL SNPs relative to their corresponding genes. Similar to our findings during the study of GR mediated PGx-eQTLs [19], the majority of the SNPs were located in non-coding regions (98%) (Fig. 2b). Among those PGx-eQTLs, approximately equal numbers of SNPs were located upstream (43.3%) or downstream (43.0%) of genes, while the remaining 11.7% were located in untranslated regions of genes, including introns (10.4%), 5’-UTRs (0.67%) and 3’-UTRs (0.67%) (Fig. 2b). There were only 5 non-synonymous SNPs in their corresponding PGx-eQTL genes, and all 5 were benign based on Clinvar. There were also 20 SNPs that resided in the promoter regions (TSS-3000 bp to 5’-UTR) of 18 corresponding genes, likely affecting transcription initiation. We questioned whether most of the SNPs function as a result of changing transcriptional activities of enhancer elements near by the genes. By comparing epigenetic markers from multiple tissues, Ernst et al. imputed the possible epigenetic status of common SNPs using the ChromHMM program [34, 35]. Therefore, we extracted those SNP-specific cis-regulatory elements from the HaploReg website [33]. 80% of all the PGx-SNPs were matched to ChromHMM annotated SNP data, among which, 50% overlapped with at least one AR ChIPseq peak. Analyzed by sectional location of the SNPs relative to the corresponding gene, roughly 80% of the matched PGx-eQTLs were located within enhancer regions, corresponding to ChromHMM states 6_EnhG and 7_Enh, regardless of their distance from the genes. In addition, TSS/Promoter status was more enriched at the start site of the gene, corresponding to ChromHMM states 1_TssA, 2_TssAFlnk and 3_TxFlnk (Fig. 2c).

In order to validate the robustness of our PGx-eQTL signals, we’ve validated top PGx-eQTL signals identified from our RNAseq experiments using qRT-PCR experiments in another independent panel of five AR-expressing LCL cell lines. Although the number of signals that can be validated in this validation panel is limited due to the availability of homozygous minor allele genotypes, we have successfully replicated two thirds of the selected signals from both DHT and Enz-treated samples, as shown in Additional file 1: Fig. S6 and Additional file 2: Table S1, which further supported the robustness of our PGx-eQTL signals.

In summary, we identified genomewide PGx-eQTL signals in response to AR-targeting drugs and specific to AR-mediated signals after multiple filtering steps and characterized potential regulatory mechanisms by SNP-based Chromatin state analysis.

### AR mediated PGx-eQTL signals were associated with breast cancer prognosis and post-treatment hormone level

**Fig. 3:**
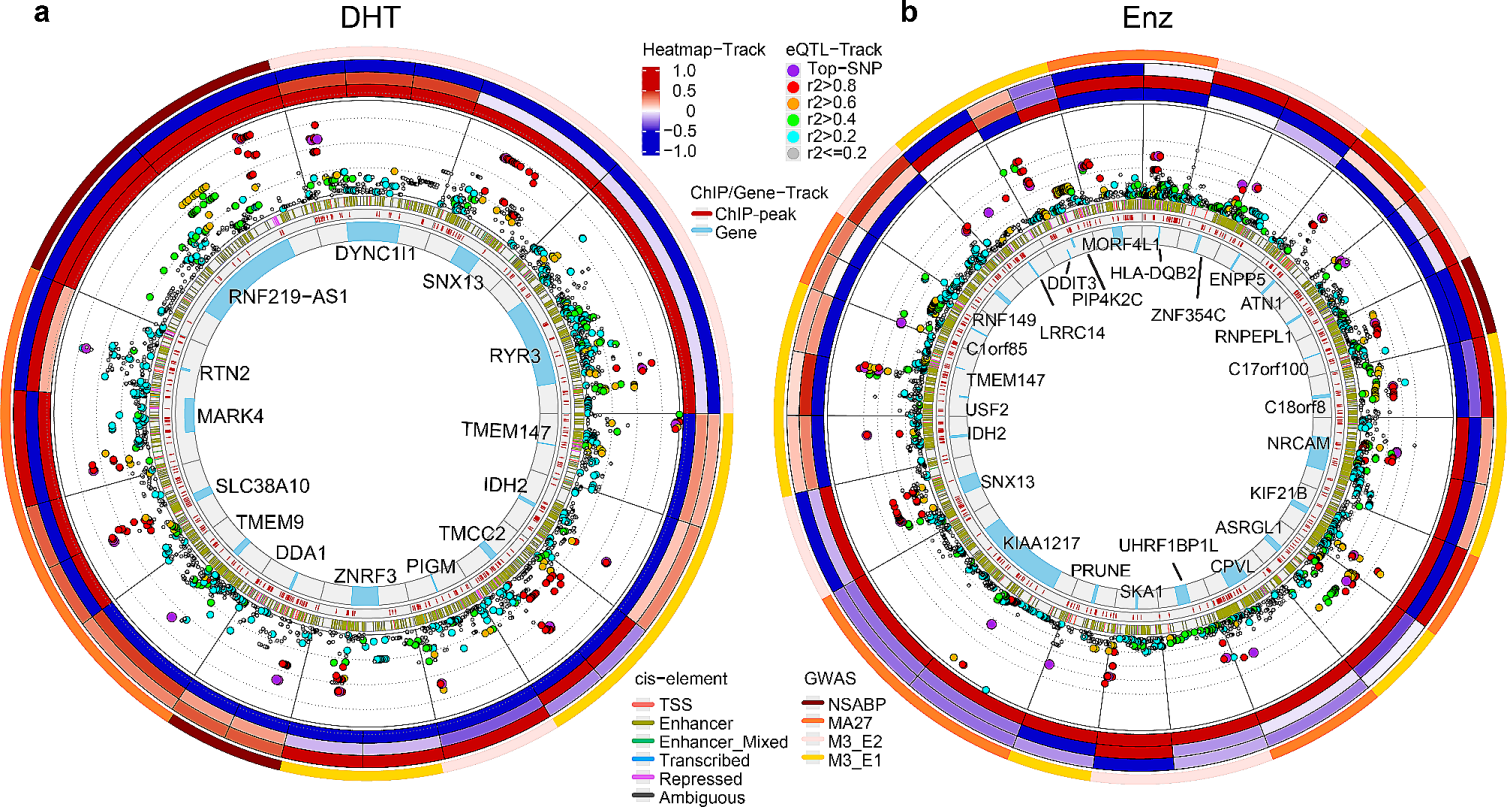
Circos plots for (**a**) DHT- and (**b**) ENZ-induced PGx-eQTL implicated in breast cancer GWAS. Each section represents a PGx-eQTL locus. Wherever more than one PGx-eQTL genes reside in the same loci, top signal was defined as the smallest p-value SNP within ChIPseq-identified binding sites among all signals within that section. Layers from outer to inner are 1st) GWAS study associated with the top PGx-eQTL signal of the section; 2nd) (3 rows) Heatmaps of relative gene expression log2-fold changes (drug vs. vehicle) for each of the three genotypes of the top SNP(WT/WT, WT/Alt, Alt/Alt); 3rd) P-values of PGx-eQTL analysis. The X-axis represents genomic coordinates and the y-axis represents –log_10_(p-value) with each log scale marked by a dashed line. Each dot represents one SNP that is PGx-eQTL to the gene of this section. The top SNP (smallest p-value SNP residing within ChIPseq identified binding sites) is colored in purple, and the rest of the SNPs were colored based on their genotyping correlation with the top SNP; 4th) ChromHMM annotation of all SNPs of the loci; 5th) AR ChIPseq peaks; 6th) Location of the PGx-eQTL gene

AR is commonly expressed in breast cancer tissues, especially in ER-positive breast cancer, but the roles that AR plays are controversial [6, 7]. We proceeded to test whether any of the AR-mediated PGx-eQTL SNPs might contribute to BC prognosis and/or endocrine therapeutic response by examining the association of these PGx-eQTL SNPs with BC phenotypes from three genome-wide association studies (GWAS) that we have published: (1) The National Surgical Adjuvant Breast and Bowel Project (NSABP) is a clinical trial of SERMs (tamoxifen or raloxifene) in the reduction of risk for developing breast cancer in women at increased risk of the disease [2]; (2) The MA27 trial is the largest trial in which efficacies of different AIs as adjuvant therapy for ER-positive breast cancer post- surgical resection of the tumor [1] was determined and (3) The Mayo-MD Anderson-Memorial Sloan Kettering (M3) trial of anastrozole as adjuvant therapy in postmenopausal women with resected early-stage breast cancer using the response phenotype of on-anastrozole plasma levels of E2 and E1 [5]. To improve the SNP coverage in LCLs, we took all of the genotyped SNPs as well as any SNPs with linkage disequilibrium (LD) coefficient r^2^ ≥ 0.8 in European population to overlap with our clinical GWAS results (SNPs with *P* < 0.005). Using these criteria, we were able to identify 13 DHT-specific PGx-eQTL loci (Fig. 3a) and 23 Enz-specific PGx-eQTL loci (Fig. 3b), corresponding to totally 45 SNPs and 36 genes, that were associated with at least one of these GWAS phenotypes. These PGx-eQTL and their associated GWAS phenotypes were summarized in Additional file 2: Table S2. To test whether the PGx-eQTL signals were specifically enriched in the BC GWAS signals or out of randomness, we further performed genomic region enrichment analysis. As shown in Additional file 2: Table S3, the PGx-eQTL signals were significantly enriched in all the GWAS datasets we have tested, with odds ratio ranges from 3.1 to 6.0 and p-values from 1.36 × 10^− 5^ to 4.19 × 10^− 45^. The most significantly enriched GWAS phenotype was post-AI level of E2, which is particularly interesting because of the relationship between androgen and estrogen biosynthesis pathway.

**Fig. 4:**
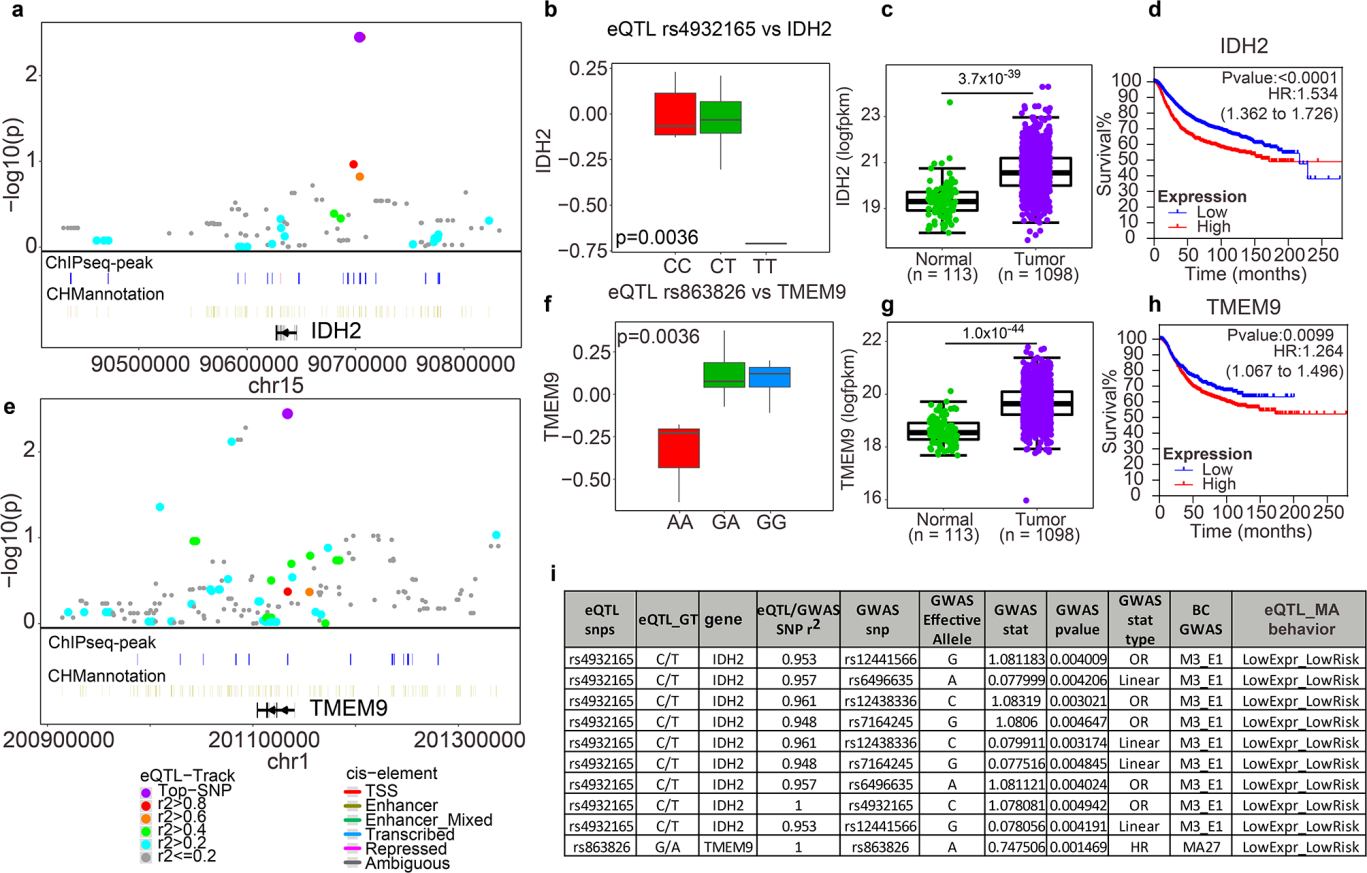
rs4932165-IDH2 and rs863826-TMEM9 PGx-eQTL loci were implicated in GWAS of post-aromatase inhibitor estrogen level and prognosis. **a, e** Locus zoom of **a** IDH2 and **e** TMEM9 loci, respectively. PGx-eQTL was presented as –log_10_(p-value) against genomic coordinates. The top SNP (rs4932165 for IDH2 and rs863826 for TMEM9) are colored purple, and the rest of the SNPs are colored based on their genotyping correlation with the top SNP. AR ChIPseq peaks and ChromHMM annotation of all SNPs of the loci are labeled under respective SNP. **b, f** DHT induced log fold change (y-axis) of **b** IDH2 and **f** TMEM9 expression by genotype of rs4932165 and rs863826, respectively. **c, g** Comparison of expression between tumor and tumor-periphery normal tissue of **c** IDH2 and **g** TMEM9 from TCGA breast cancer cohort. Statistical significance was tested by mann-whitney’s nonparametric test. **d, h** Relapse free survival analysis of multiple cohorts of breast cancer, grouped by expression of **d** IDH2 and **h** TMEM9. **i** List of PGx-eQTL SNPs and GWAS signals that is either exactly match or in LD (r^2^ > 0.8) of IDH2 and TMEM9 loci

Among these loci were some well-studied cancer related genes such as IDH2, a mitochondrial-located citric cycle enzyme, that has been reported to be frequently mutated and involved in the metabolomic reprogramming of multiple cancers [40–42], and TMEM9, a wnt-signalling amplifier that promotes v-ATPase assembly and accelerates APC degradation [43]. Regional plots of these two loci are presented in Fig. 4a and e, respectively. As shown in Fig. 4b, the SNP rs4932165 T/T genotype corresponds to DHT-induced downregulation of IDH2, and is correlated with lower post-AI E2 levels (Fig. 4i) which have been associated with better response to anastrozole treatment [44]. In fact, IDH2 is significantly lower expressed in normal tissue compared to tumor (Fig. 4c), and lower IDH2 expression is associated with better relapse-free survival in other breast cancer cohorts (Fig. 4d). Therefore, patients with T/T genotypes at rs4932165 might benefit from increased AR activity either resulting from increased androgen levels after AI therapy or might be from direct AR-agonist treatment.

In a similar fashion, the SNP rs863826 A/A genotype was associated with DHT-induced downregulation of TMEM9 (Fig. 4f) and with reduced risk of relapse in the MA27 BC cohort who received AI therapy (Fig. 4l). TMEM9 expression was also lower in in normal compared to tumor tissue (Fig. 4g), and lower expression of TMEM9 was associated with better prognosis when given AI therapy. (Fig. 4h). Therefore, patient with the A/A genotype at rs853826 might benefit from increased androgen levels, either resulting from inhibited estrogen biosynthesis by AIs or by direct AR-agonist treatment.

### AR mediated PGx-eQTL signals were implicated in breast cancer risk and other GWAS phenotypes

**Fig. 5:**
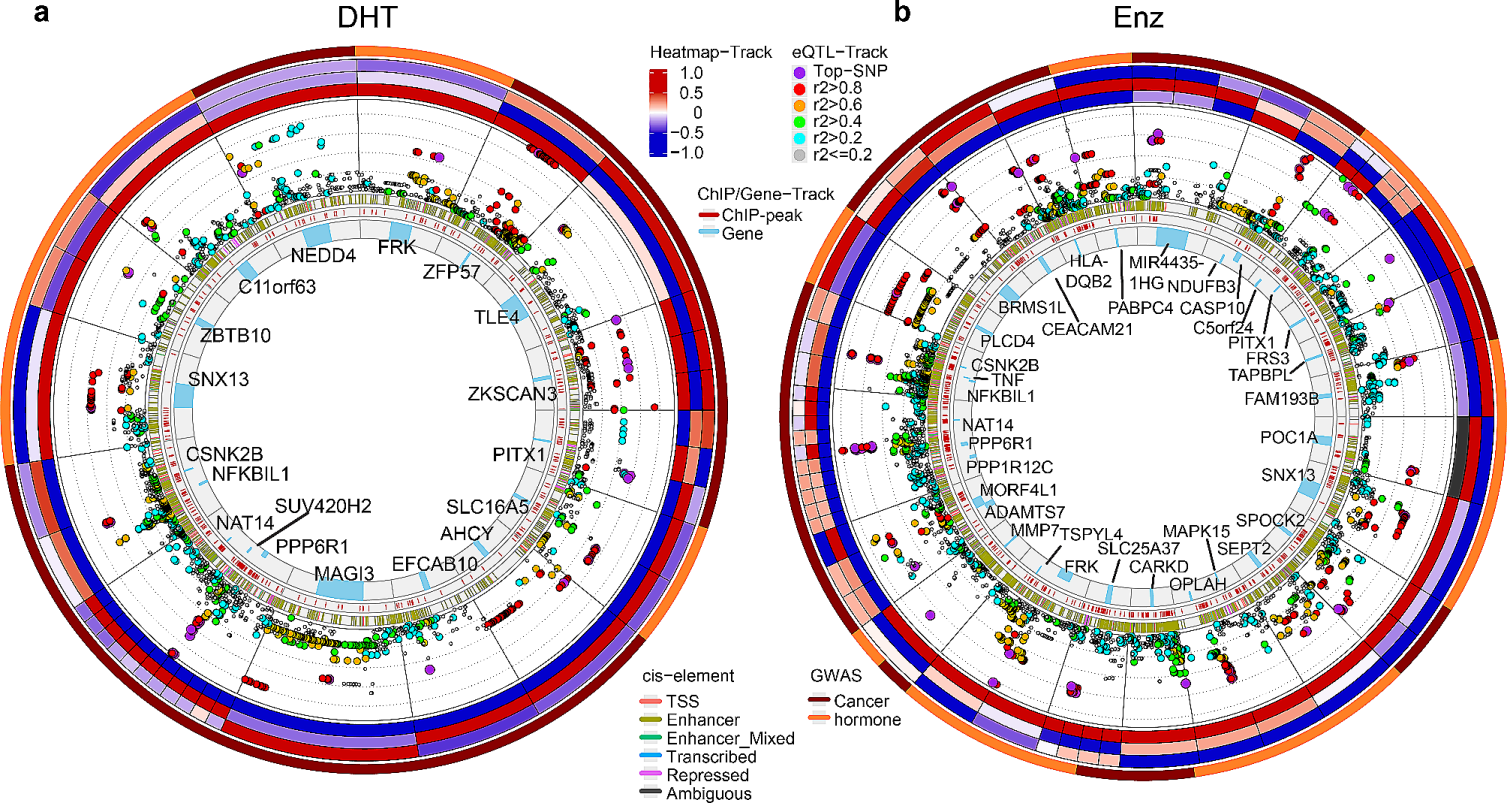
Circos plots for (**a**) DHT and (**b**) ENZ induced PGx-eQTL implicated in GWAS catalog database. Each section represents a PGx-eQTL locus. Wherever more than one PGx-eQTL genes reside in the same loci, the top signal was defined as the smallest p-value SNP residing within ChIPseq identified binding sites among all signals of the section. Layers from outer to inner are 1st) GWAS study type (cancer or hormone-related) implicated by the top PGx-eQTL signals within that section; 2nd) (3 rows) Heatmaps of relative gene expression log2-fold changes (drug vs. vehicle) for each of the three genotypes of the top SNP; 3rd) P-values of PGx-eQTL analysis. The X-axis represents genomic coordinates and the y-axis represents –log_10_(p-value) with each log scale marked by a dashed line. Each dot represents one SNP that is PGx-eQTL to the gene within this section. The top SNP (smallest p-value SNP residing within ChIPseq identified binding sites) is colored as purple, and the rest of the SNPs were colored based on their genotype correlation with the top SNP; 4th) ChromHMM annotation of all SNPs of the loci; 5th) AR ChIPseq peaks; 6th) Location of the PGx-eQTL gene

In the previous section, we primarily investigated potential association between AR PGx-eQTLs and prognosis of endocrine therapies in breast cancer using data from three BC GWAS we published. In order to identify additional phenotypes that AR-mediated PGx-eQTL signals might be involved in, we performed overlapping analysis between AR-mediated PGx-eQTLs and all GWAS signals downloaded from the GWAS catalog database [25]. Similar to our analysis using the BC GWAS, we also included SNPs that were r^2^ ≥ 0.8 to PGx-eQTL signal SNPs. These PGx-eQTL genes mapped widely to a number of different traits in almost every aspect of human health, specifically in tissues that has been significantly attributed to androgen and AR signalling, such as cholesterol metabolism, various neuropsychiatric disorders, immune regulation, and skeletal muscle development (Additional file 1: Fig. S7, Additional file 2: Table S4). Since we were particularly interested in the function of AR in association with cancer and sex hormone levels and regulation, we specifically extracted GWAS traits that were related to either cancer or sex hormone levels and the results are summarized in Additional file 2: Table S5. This analysis identified another 15 DHT-induced PGx-eQTL loci and 23 Enz-induced PGx-eQTL loci, corresponding to 54 SNPs and 43 genes, respectively. Five loci were shared between DHT and Enz loci, and three loci were shared with BC GWAS identified in previous section. The eQTL analysis, aligned with ChIPseq, ChromHMM annotation for each locus, are presented as the circos plots shown in Fig. 5. The identified PGx-eQTL and their associated GWAS were summarized in Additional file 2: Table S6. Similar to breast cancer GWAS, genomic region enrichment was performed for PGx-eQTL loci against GWAS loci from GWAS Catalogue. Odds ratio of 3.1 and 3.8 with p values 1.78 × 10^− 16^ and 1.98 × 10^− 15^ were found for cancer and hormone GWAS loci, respectively, indicated specific enrichment of potentially functional variants in these loci (Additional file 2: Table S7).

**Fig. 6:**
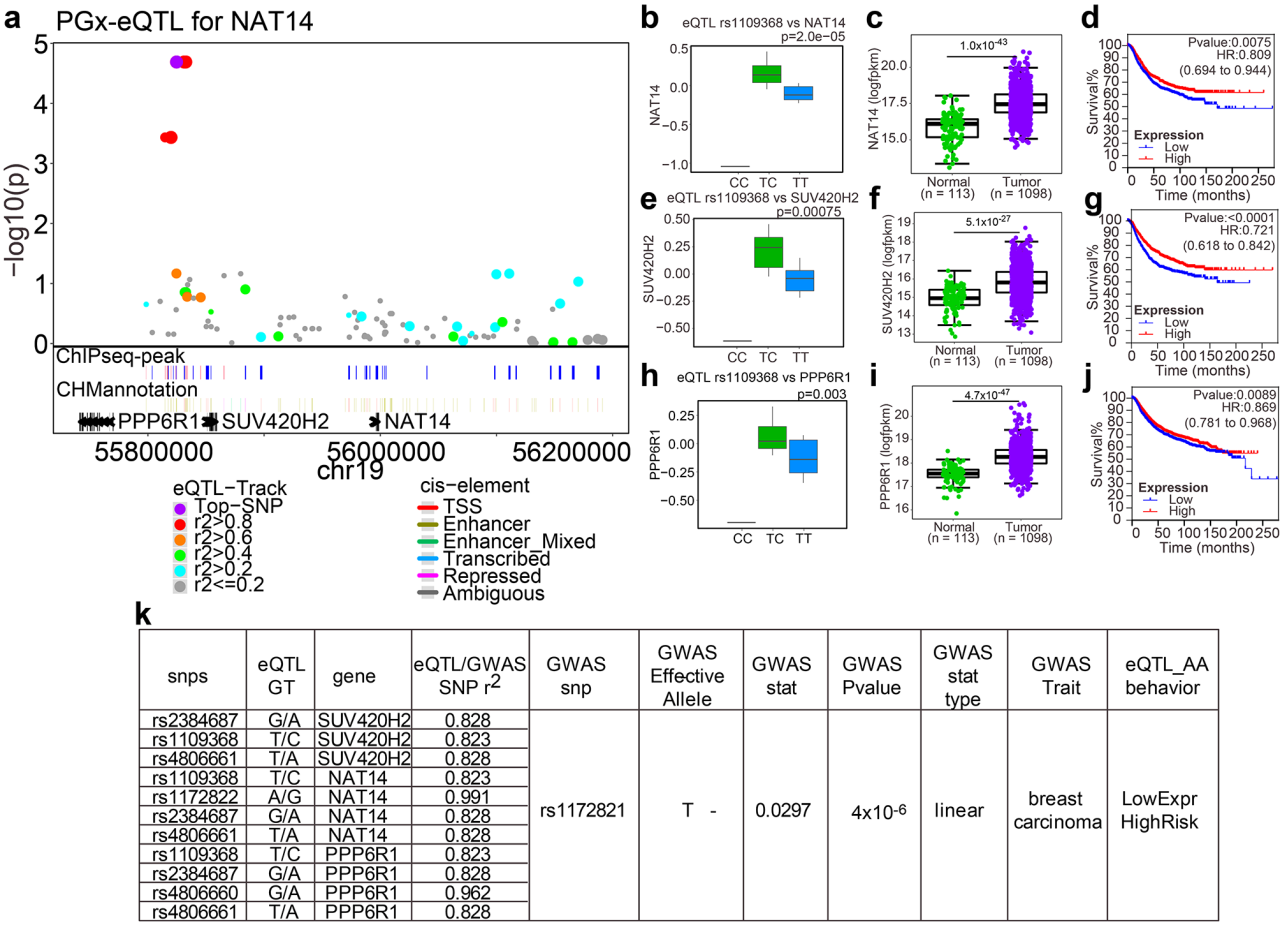
NAT14-SUV420H2-PPP6R1 Loci was implicated in GWAS of breast cancer. **a** Locus zoom for NAT14. PGx-eQTL was presented as −log10(p-value) against genomic coordinates. The top SNP (rs1109368) are colored purple, and the rest of the SNPs are colored based on their genotyping correlation with the top SNP. AR ChIPseq peaks and ChromHMM annotation of all SNPs of the loci are labeled under respective SNP. **b**, **e**, **h** DHT induced log fold change (y-axis) of **b** NAT14, **e** SUV420H2, and **h** PPP6R1 expression by genotype of rs1109368, respectively. **c**, **f**, **i** Comparison of expression between tumor and tumor-periphery normal tissue of **c** NAT14, **f** SUV420H2, and i PPP6R1 from TCGA breast cancer cohort. Statistical significance was tested by Mann-Witney’s nonparametric test. **d**, **g**, **j** Relapse-free survival analysis of multiple cohorts of breast cancer, grouped by expression of d NAT14, **g** SUV420H2, and **j** PPP6R1, respectively. **k** List of PGx-eQTL SNPs and GWAS signals that are either exact matching or within LD (r^2^>0.8) of NAT14/SUV420H2/PPP6R1 locus

One of the most interesting loci was the PPP6R1-SUV420H2-NAT14 locus on chromosome 19. The three genes in this locus were coexpressed (Additional file 1: Fig. S8a) at baseline and appeared to be regulated coordinately through DHT-induced expression regulation (Fig. 6b and e, 6 h) by a group of linked SNPs (Additional file 1: Fig. S8b). The locus zoom for the most significant gene, NAT14, is presented in Fig. 6a and the other genes are presented in Additional file 1: Fig. S8c, S7d. The minor alleles of these PGx-eQTL SNPs, e.g. the homozygous C-allele in rs1109368, was associated with lower expression levels of these three genes (Fig. 6b and e, 6 h) and higher risk of breast cancer (Fig. 6k). Moreover, these three genes were more lowly expressed in peripheral normal tissues compared to tumor tissue (Fig. 6c, f and i), and lower levels of expression were also associated with worse relapse free survival for breast cancer, particularly NAT14 and SUV420H2 (Fig. 6d, 6 g, 6j).

Another PGx-eQTL locus mediated by Enz involved Caspase 10 (CASP10) (Additional file 1: Fig. S9). CASP10 was upregulated by Enz in a GG-genotype of rs3769823 dependent manner (Additional file 1: Fig. S9b). The G-allele of rs3769823, or the T-allele of the linked SNP rs3679821, is also associated with reduced breast cancer risk (Additional file 1: Fig. S9e). CASP10 is more highly expressed in normal tissue compared to tumor tissue (Additional file 1: Fig. S9c), and higher expression is associated with better prognosis for breast cancer (Additional file 1: Fig. S9d). In addition to breast cancer, Caspase 10 is also associated with basal cell carcinoma, non-melanoma skin carcinoma, non-small cell lung carcinoma, prostate carcinoma, and cancer risk in general (Additional file 2: Table S5).

Finally, we found three PGx-eQTL genes that were shared between our breast cancer GWAS and the GWAS catalog traits of cancer and hormone levels, namely SNX13, HLA-DQB2, and MORF4L1. We have highlighted those genes in red in Additional file 2: Table S5. One of these three genes, SNX13 (Sorting Nexin 13), was associated with both post-AI E2 levels in the M3 cohort [5] as well as sex-hormone binding globulin levels [45], which are likely to be relevant to breast cancer. The SNX13 locus is presented in Additional file 1: Fig S10a. The homozygous minor allele genotype T/T of the PGx-eQTL SNP rs2723497 was associated with higher SNX13 levels induced by DHT (Additional file 1: Fig S10b). However, the T-allele was both associated with lower levels of baseline sex hormone-binding globulin as well as higher level of post-AI E2 levels (Additional file 1: Fig S10e). Interestingly, SNX13 was shown to be downregulated in all breast cancer subtypes, but particularly in the triple-negative subtype (Additional file 1: Fig S10c), which may result in worse prognosis for all subtypes of- breast cancer because of a higher rate of triple negative breast cancer subtype (Additional file 1: Fig S10d). DHT mediated induction of SNX13 might potentially resulted in better outcomes via reducing sex hormone-binding globulin, despite the presence of higher free plasma E2 level.

## Discussion

Understanding how TF-mediated transcriptional regulation is affected by both genetic context as well as environmental factors such as levels of endogenous hormones and drug treatment is critical for individualized medicine since the potency of the treatment may differ significantly among individuals. Following our earlier findings, both AR agonists (DHT) and antagonists (Enz) may be potentially beneficial [15] in the treatment of ER + breast cancer, depends on the AR/ER ratio in the BC cells [15]. In this manuscript, we have identified additional genetic factors that may potentially affect the treatment effectiveness of endocrine therapies including SERMs, AI, DHT and Enz. For example, we showed that DHT suppressed the expression of IDH2 in a rs4932165 T-allele dependent manner, which was also associated with lower E2 level in ER + BC patients post-adjuvant anastrozole therapy. IDH1 and IDH2 are TCA cycle enzymes which convert isocitrate to α-ketoglutarate. IDH1 is located in cytosol while IDH2 is in mitochondria. Mutation and overexpression of the IDH genes could significantly reprogram the energy metabolism of cells and possibly result in oncogenesis [42, 46, 47]. IDH1 is frequently mutated in multiple cancers, especially in low grade glioma and acute myeloid leukemia [46]. Although IDH2 genes are rarely mutated in breast cancer [40], nearly 35% of cases either have IDH2 amplification or mRNA overexpression (Fig. 4, Additional file 1: Fig. S11), and it appears that IDH2 expression is correlated with worse prognosis in primary breast cancer (Fig. 4). Gain of function of IDH genes results in reduced production of α-Ketoglutarate, a key cofactor for certain histone and DNA demethylases, thus changing the epigenetic profile of cells [48]. Moreover, mitochondria are required for the biosynthesis of all steroid hormones including androgens and estrogens [49], and a disrupted TCA cycle has been implicated in endocrine therapy resistance, recurrence and metastasis [50]. All of these mechanisms may contribute to the AI response and relative post-AI hormone levels as observed in our GWAS and survival analysis shown in Fig. 4. Another gene that is also suppressed by DHT in a genotype dependent fashion and thus is associated with worse post-AI relapse-free survival is TMEM9 (Fig. 4). TMEM9 is frequently upregulated in various types of cancer, especially breast cancer. 4% of BC tumors carry TMEM9 amplifications and nearly half are TMEM9 upregulated (Fig. 4, Additional file 1: Fig. S11b). TMEM9 promotes lysosome activation, and in turn facilitates the degradation of APC, which releases β-catenin and activates Wnt-signalling in liver cancer [51] and colorectal cancer [52]. mTOR activation also requires lysosome surface and TMEM9 has also been linked to activation of mTOR signalling, which has been associated with mammary tumorigenesis [53], consistent with our findings.

By mining the GWAS catalog database, we identified two additional loci potentially related to breast cancer risk. One was the NAT14-SUV420H2(KMT5C)-PPP6R1 locus. DHT suppressed the expression of all three genes in this locus, which appeared to be associated with higher breast cancer incidence rate and worse outcomes. This is also consistent with the finding that all three genes appear to be co-upregulated in breast cancer compared to normal tissue (Fig. 6, Additional file 1: Fig. S11c). None of these genes has been widely studied before, but one can postulate their potential roles via their reported cell functions. NAT14 is predicted to be an acetyltransferase based on sequence, and has been reported to bind DNA and play a TF-like role [54]. SUV420H2, also known as KMT5C, is a histone H4 Lys20 methyltransferase, and is reported to facilitate non-homologous end-joining (NHEJ)-directed DNA repair [55, 56]. PPP6R1 is a regulatory subunit of protein phosphatase 6 (PP6). PP6 responses to innate immune signals via the cGAS-STING pathway [57], and is implicated in tumor suppression due to its role in restricting cell cycle progression [58].

The other locus of interest is CASP10, suppressed by Enz in a rs3769823 genotype dependent manner. Consistently, CASP10 is frequently downregulated in breast cancer, and overexpression of CASP10 appeares to improve the overall prognosis in breast cancer (Fig. S6d), which indicates a better prognosis if being treated by Enz. Caspase 10 is a part of the apoptotic cascade in response to extrinsic death signals upstream of caspase 3 and 7. It has been widely studied in cancer mechanisms and therapeutics [59, 60]. Caspase 10 sensitizes BC cells to TRAIL-induced apoptosis [61], and is required for therapeutic effect of taxane [62]. It is not surprising if it is also involved in cellular response to Enz similar to what is shown in this manuscript.

Although mammary tissue is the most relevant tissue for this study, there is not a panel of mammary cells we can use to perform this study. Despite that cancer cell lines have been widely used in studying drug response, their genomic alteration (duplicated, deleted, fused, etc.) and the nature of heterogeneity have prevented them from being employed in the study of inheritable SNP-dependent responses to natural ligands and drugs such as those used in this study. As an alternative model system, the LCL panel employed in this study has been extensively characterized by our group and others. The LCL panel represents a wide variety of human genomic diversity and has been employed in many GWAS, especially for context-dependent drug response [23, 63, 64]. In order to improve the specificity of AR eQTL-SNPs, we chose to perform our experiments using 15 AR-expressed and 15 AR-null cell lines, which enabled us to remove AR-independent signals. To further ensure that the PGx-eQTL signals were in response to either AR agonist or antagonist, we further removed any signals that were not reversed by DHT/Enz dual-treatments compared to either treatment alone, and the remaining signals were considered to be AR agonist/antagonist-inducible PGx-eQTL signals. Another limitation of the study is the relatively small sample size. eQTL analysis heavily relies on large sample sizes and high SNP allele frequencies to achieve highly significant p-values. The relatively small sample size limited our capability to detect genome-wide significant signals. Therefore, instead of relying only on statistically defined p-values, we chose to cross-validate our signals with the biologically informative datasets. We carefully filtered our PGx-eQTL signals with ChIPseq experimentally validated AR binding sites in breast and prostate tissues and examined clinical relevance using breast cancer GWAS data, which allowed us to identify more functional signals. We have previously employed a similar strategy in our PGx-eQTL study of glucocorticoid receptor analysis and identified highly clinically relevant signals [19]. Most of the GWAS signals are located in the non-coding regulatory regions without understanding how these SNPs might contribute to gene function and disease risks or prognosis. Our approach has identified the potential biological and clinical relevance of PGx-eQTL signals that have been further validated from the results of GWAS of breast cancer risk or prognosis. These SNPs likely impact disease or treatment response through their effect on gene expression in the presence of different chemical compounds, a unique phenomenon that should be considered in future studies.

## Conclusions

In conclusion, we performed genomewide PGx-eQTL analysis using agonists and antagonists of AR and identified DHT or Enz – AR regulated genes that are potentially influential in the endocrine therapy of breast cancer, genes and SNPs that could potentially serve as biomarkers for individualized endocrine therapy of breast cancer.

### Electronic supplementary material

Below is the link to the electronic supplementary material.

Supplementary Material 1

Supplementary Material 2

## Data Availability

The datasets generated and/or analysed during the current study are available on GEO accession GSE245417.Codes used for the primary analysis and visualization of this work has been deposited to github repository: @huanyaogao/Pharmacogenomic-eQTL.

## Abbreviations

DHT: Dihydrotestosterone
Enz: Enzalutamide
AR: Androgen receptor
ER: Estrogen receptor
GR: Glucocorticoid receptor
PGx: Pharmacogenomic
eQTL: Expression quantitative trait loci
ChIP-seq: Chromatin immunoprecipitation - sequencing
GWAS: Genome-wide association study
SNP: Single nucleotide polymorphism
NSABP: National Surgical Adjuvant Breast and Bowel Project
SERM: Selective estrogen receptor modulator
AI: Aromatase inhibitors
BC: Breast cancer
TNBC: Triple negative breast cancer
TF: Transcription factor
