# Supplementary material for: Androgen receptor-mediated pharmacogenomic expression quantitative trait loci: implications for breast cancer response to AR-targeting therapy
Source: Breast Cancer Res. 2024 Jul 4;26:111. doi: 10.1186/s13058-024-01861-2 (PMC11225427; doi:10.1186/s13058-024-01861-2)
Supplement: Supplementary file 1 — Supplementary Material 1 [file 13058_2024_1861_MOESM1_ESM.docx]

**Supplementary Figures**


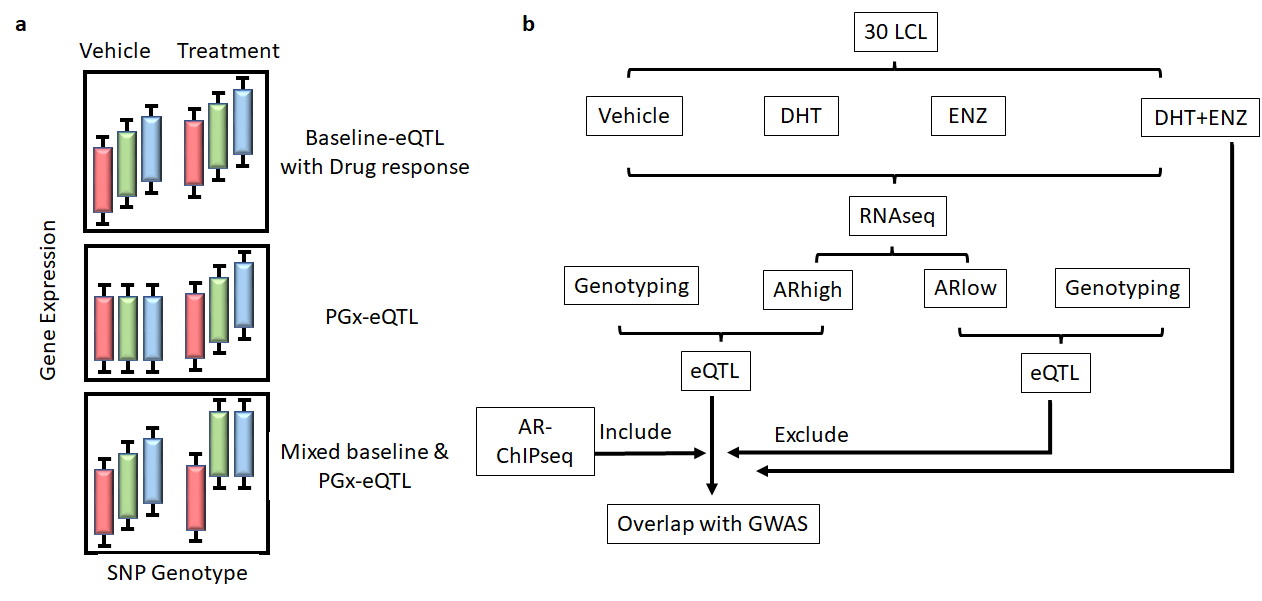


**Supplementary Figure S1: Experimental design of pharmacogenomic-expression quantitative trait loci (PGx-eQTL) study of androgen receptor. a** Simplified representation of PGx-eQTL. PGx-eQTL refers to genotype-specific changes in gene expression upon drug treatment, whereas baseline eQTL refers to genotype-specific variation of gene expression regardless of drug treatment. Certain genes can be regulated in both ways and exhibit mixed eQTL phenotype. **b** AR PGx-eQTL study pipeline. 30 LCLs, including 15 with AR expressed and 15 with no AR expression (AR-null), were treated with AR agonist (DHT), antagonist (Enzalutamide, ENZ), a combination of DHT and ENZ, and vehicle, and submitted for RNAseq, which was combined with genotyping data to identify PGx-eQTL signals. The AR or AR-targeting ligand/drug independent signals were further identified by signals from AR-null cells, and those not-reversible by the antagonist (DHT by ENZ or vice versa), thus were excluded. Finally, resulted signals were overlapped with AR CHIPseq binding peaks and then overlapped with GWAS data.


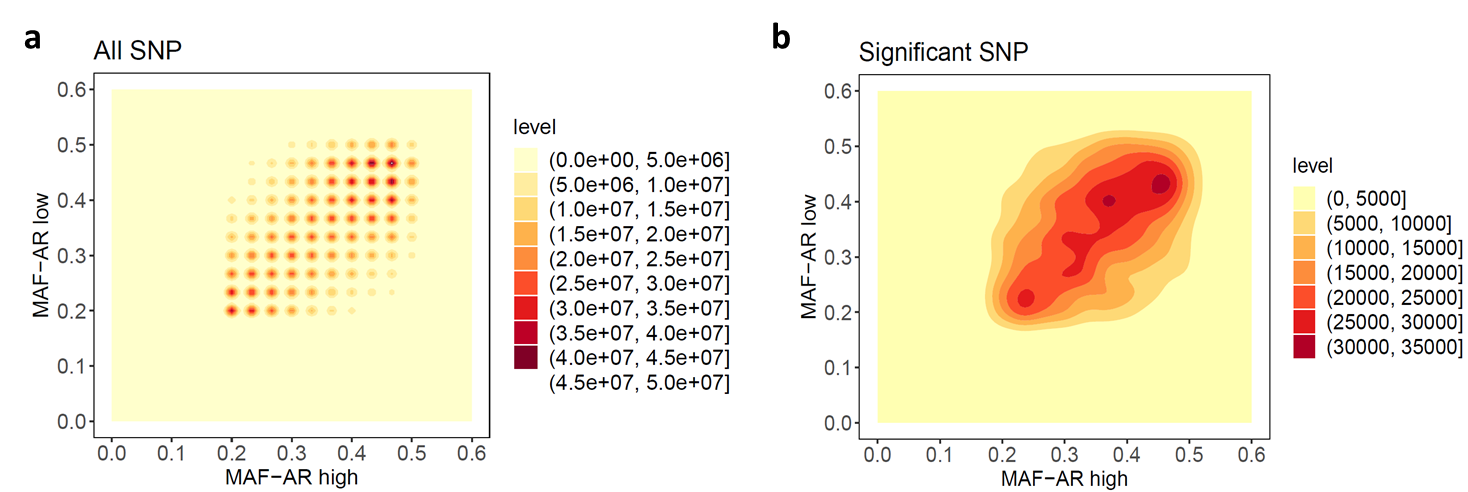


**Supplementary Figure S2: Contour plots comparing MAFs of either all SNPs (a) and SNPs with significant PGx-eQTL signals (b) between AR-expressed and AR-null cells.** Color ramp from yellow to maroon represent low to high number of SNPs with specific MAF in corresponding AR-high and AR-null cells, respectively.


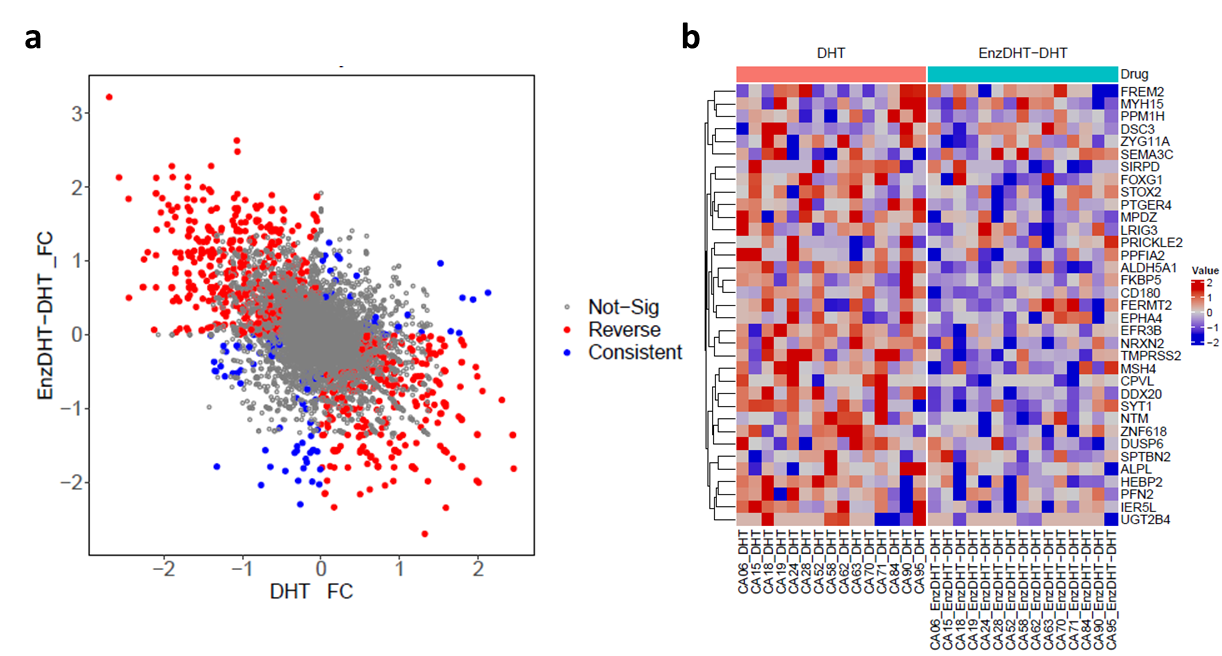


**Supplementary Figure S3: Differential expression analysis (a)** Log2 fold change between DHT and vehicle treated samples (x-axis) vs between Enz-DHT dual-treated and DHT-only treated samples (y-axis). Each dot represent a gene and p-values < 0.01 were considered significant and was colored based on the directionalities of the two pairs of comparisons.  **(b)** Heatmap of log2 fold-changes between DHT and vehicle treated samples and between Enz-DHT dual-treated and DHT-only treated samples of some classic AR-downstream genes expressed in LCLs.


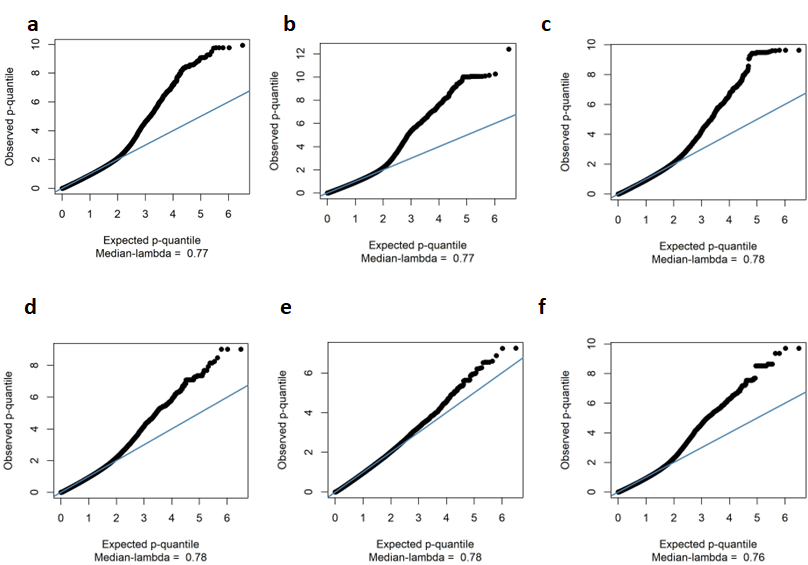


**Supplementary Figure S4: QQplots for PGx-eQTL analysis. a-c**, AR-expressed cells and **d-f** AR-null cells from. **a,d** DHT-induction; **b,e** ENZ-induction; and **c,f** DHT/ENZ double-treatments.


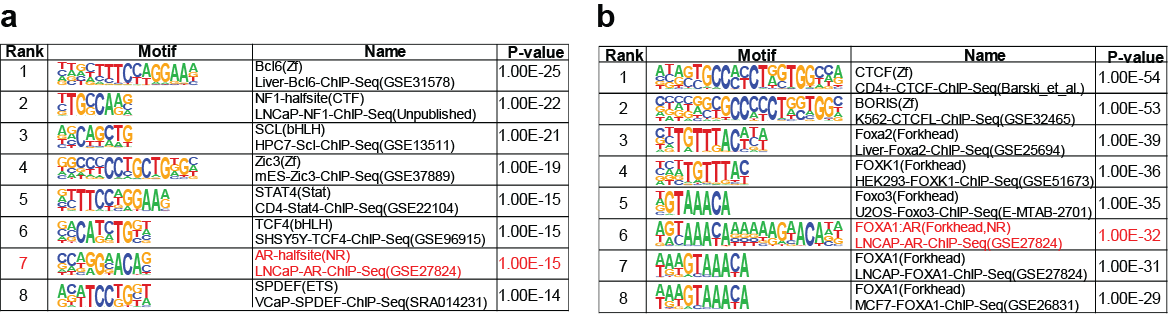


**Supplementary Figure S5 Drug-specific AR PGx-eQTL signals motif analysis.** **a,b** Homer motif analysis of SNP periphery sequences from **a** DHT **b** ENZ-specific induced PGx-eQTL signals, filtered by AR ChIPseq binding sites. Classical AR binding motif was highlighted in red


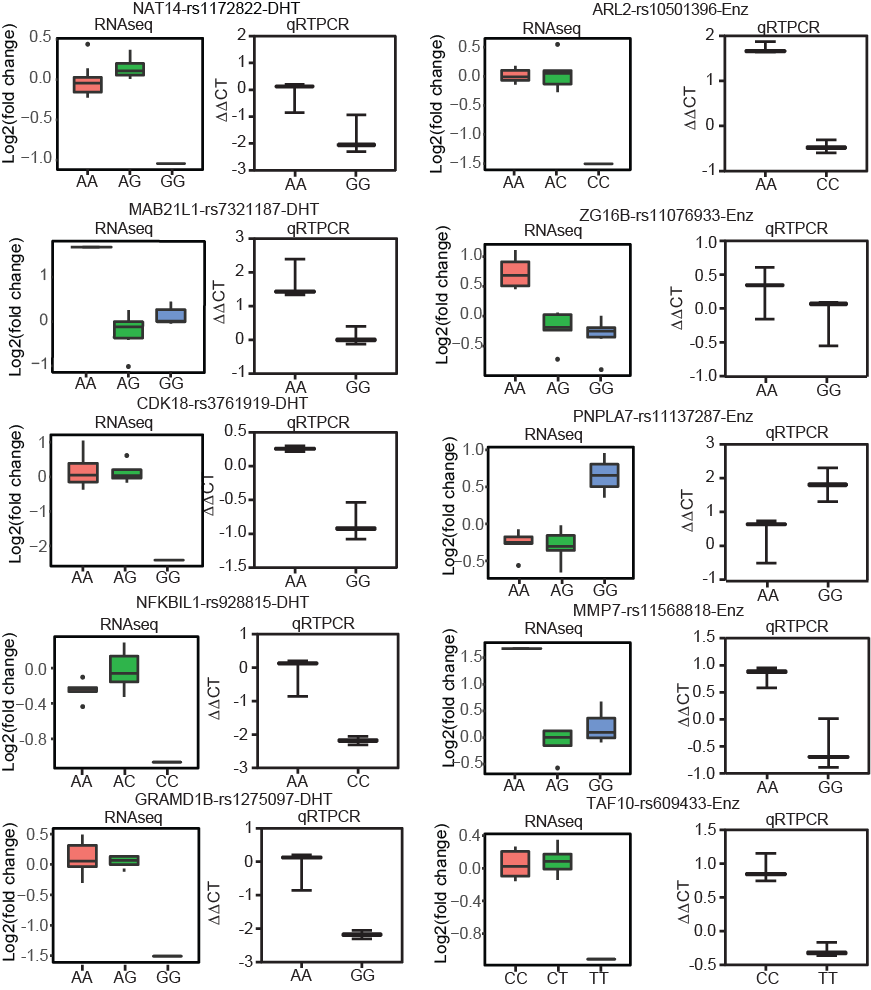


**Supplementary Figure S6: qRTPCR validation using independent cohort of LCL panel were aligned side-by-side with top PGx-eQTL signals.** PGx-eQTL identified by RNAseq were presented as boxplot by genotypes (box-inner quartiles, whiskers-10-90% of data). Y-axis represents log2(fold-changes) of gene expression (tpm) in DHT/Enz treated sample compared with vehicle treated samples. qRT-PCR was performed on an independent cohort of cell lines with homozygous WT or alternative alleles on corresponding SNPs, and were presented as boxplots (box-inner quartiles, whiskers-full range of data). Y-axis represents ΔΔCT values of DHT/Enz treated sample compared with vehicle treated samples.


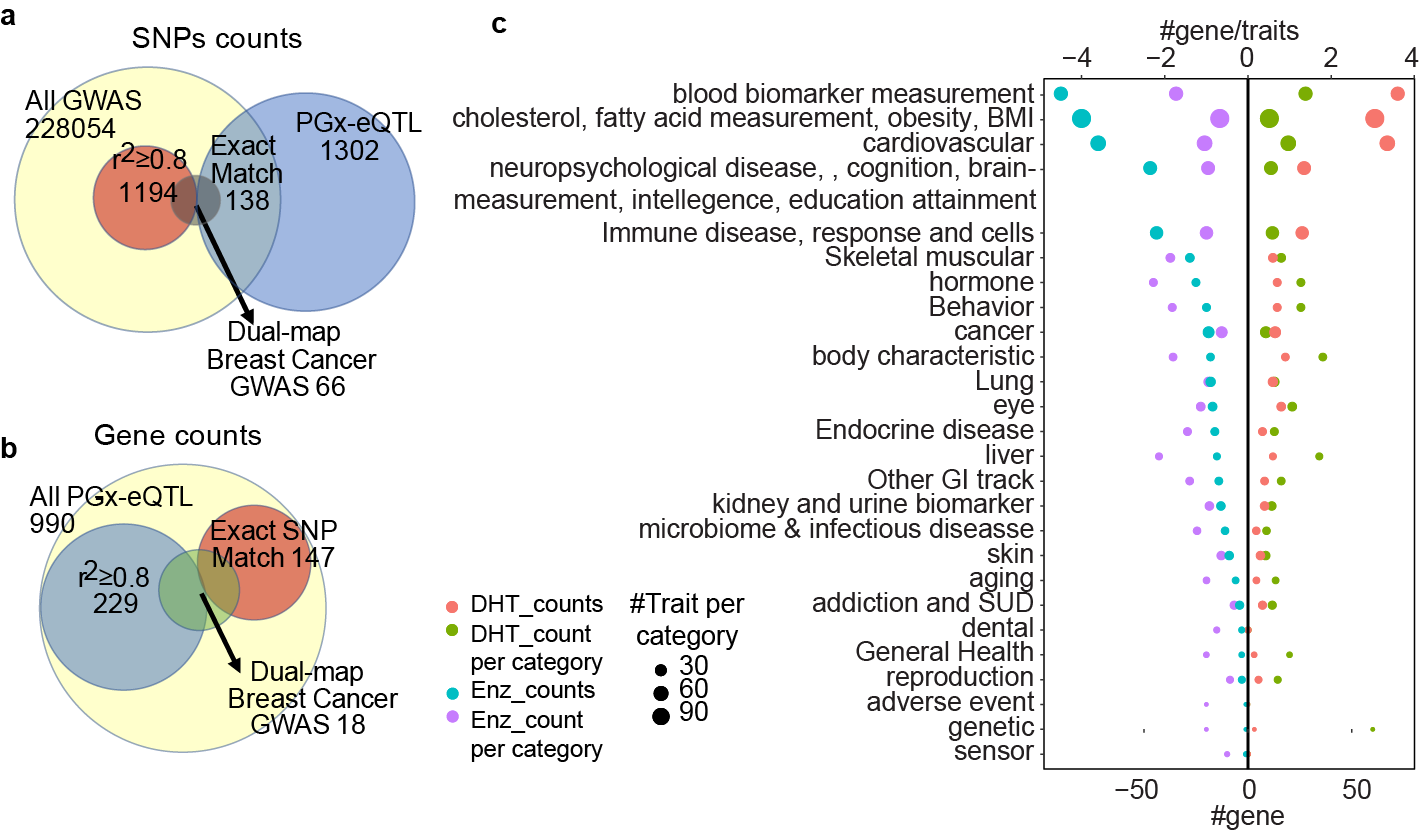


**Supplementary Figure S7: Summary of AR-mediated PGx-eQTL overlapped with GWAS catalog SNPs. a** Counts of SNPs from all GWAS catalog signals, all PGx-eQTL signals, exact match SNPs between GWAS and PGx-eQTL, GWAS and PGx-eQTL SNPs that are in same LD block (r^2^≥0.8), and SNPs that are mapped to both breast cancer GWAS and GWAS catalog signals. **b** Counts of genes from all PGx-eQTL signals, genes with exact matching SNPs between GWAS and PGx-eQTL, genes with GWAS and PGx-eQTL SNPs that are in same LD block (r^2^≥0.8), and genes with SNPs that are mapped to both breast cancer GWAS and GWAS catalog signals. **c** Summary of number of genes (red, blue, bottom axis) or number of genes per trait (green, purple) within category of GWAS phenotypes identified by PGx-eQTL and GWAS catalog overlapping analysis.


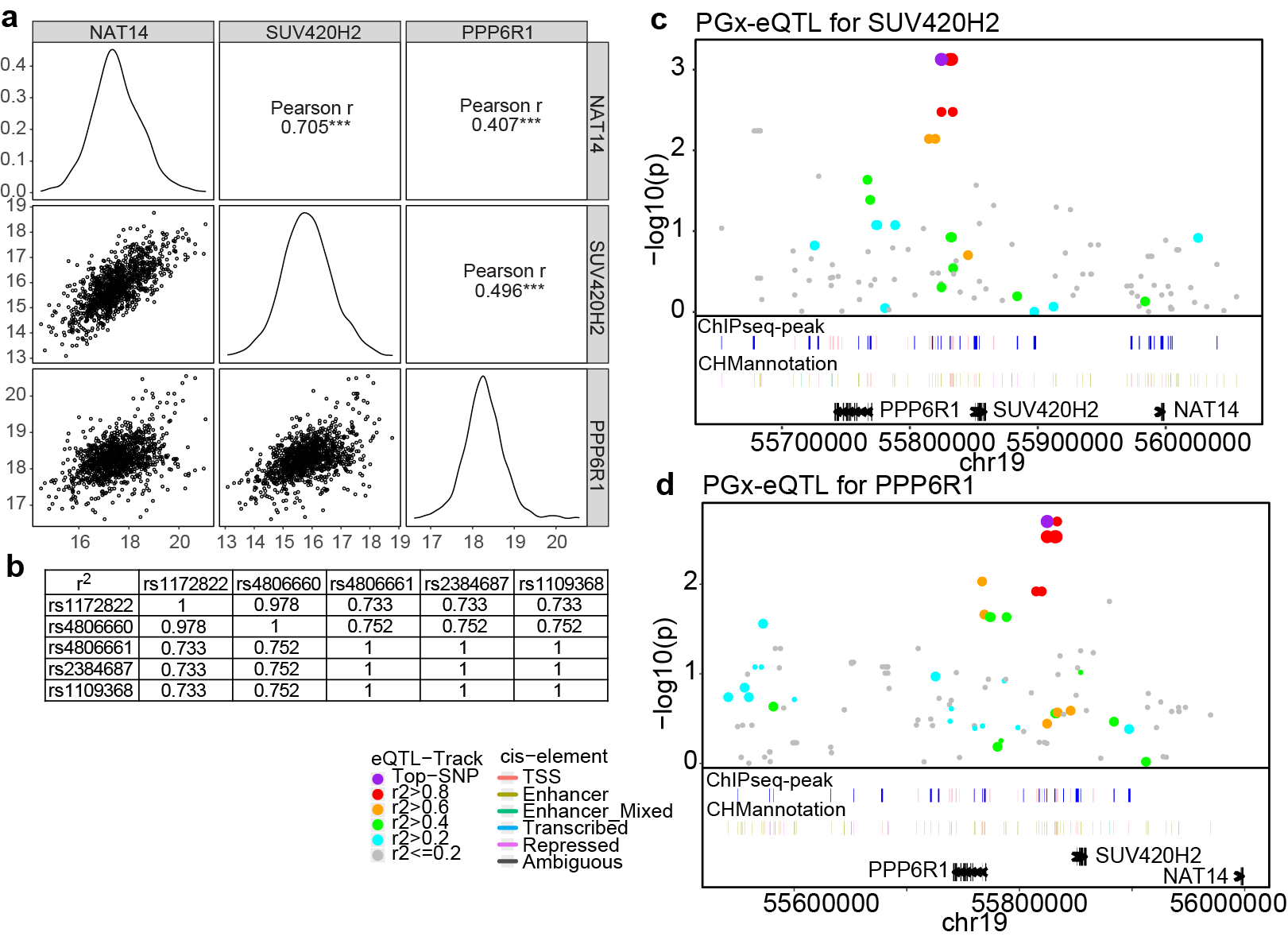


**Supplementary Figure S8: NAT14-SUV420H2-PPP6R1 Loci genes and SNPs correlation and locus zoom. a** Expression correlation of NAT14, SUV420H2, and PPP6R1 in the TCGA-breast cancer cohort. Expression distribution was displayed at the diagonal, pearson correlation coefficient was labeled at the cross of the gene pair and scattered plots of normalized expression of the two genes is on the other side of cross plot. **b** Correlation (r^2^) of the PGx-eQTL SNPs within the NAT14-SUV420H2-PPP6R1 locus. **c,d** Locus zoom for **c** SUV420H2 and **d** PPP6R1, respectively. PGx-eQTL was presented as –log_10_(p-value) against genomic coordinates. The top SNP (rs1109368 for SUV420H2 and rs4806660 for PPP6R1) are colored purple, and the rest of the SNPs are colored based on their genotyping correlation with the top SNP. AR ChIPseq peaks and ChromHMM annotation of all SNPs of the loci are labeled under respective SNP.


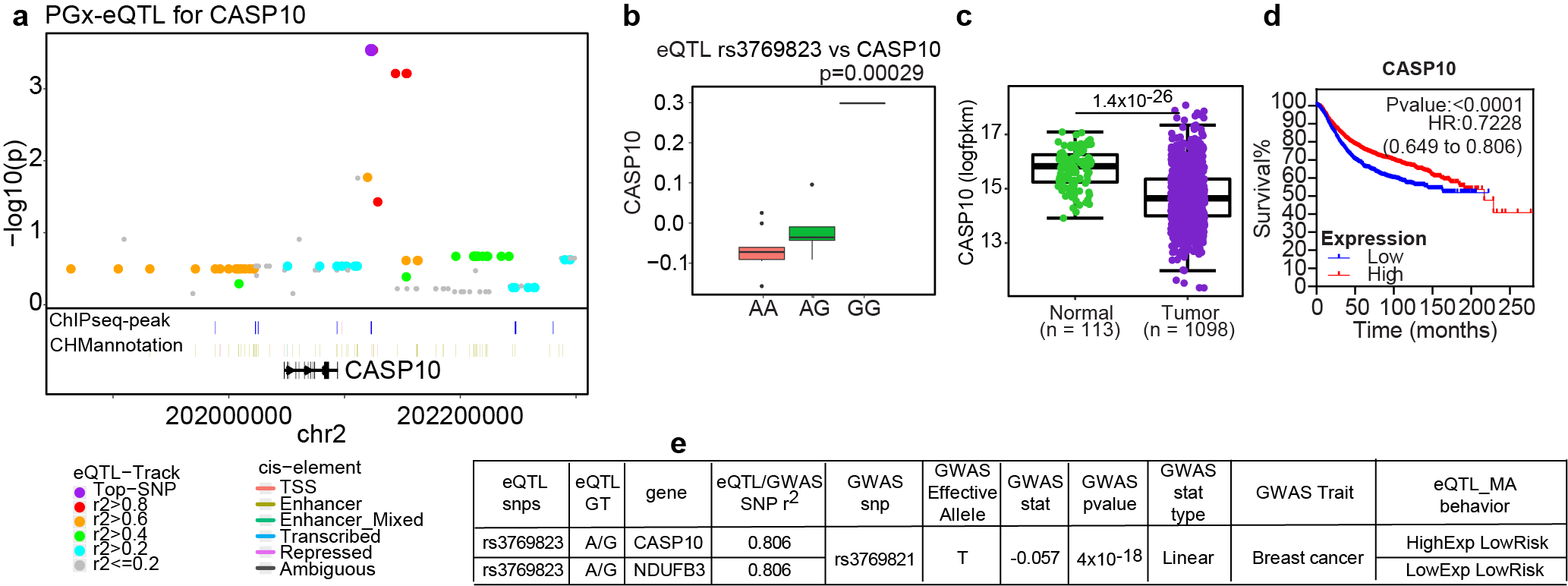


**Supplementary Figure S9: rs3769823-CASP10 Loci was implicated in GWAS of breast cancer risk. a Locus zoom of rs3769823-CASP10 loci.** Enz mediated PGx-eQTL analysis for CASP10 was presented as –log_10_(p-value) against genomic coordinates. The top SNP (rs3769823) is colored purple, and the rest of the SNPs are colored based on their genotyping correlation with the top SNP. AR ChIPseq peaks and ChromHMM annotation of all SNPs of the loci are labeled under respective SNP. b Enz induced log fold change (y-axis) of CASP10 expression by genotype of rs3769823. c Comparison of expression between tumor and tumor-periphery normal tissue of CASP10 from TCGA breast cancer cohort. Statistical significance was tested by mann-whitney’s nonparametric test. d Relapse free survival analysis of multiple cohorts of breast cancer, grouped by expression of CASP10. e List of PGx-eQTL SNPs and GWAS signals that is either exactly match or in LD (r^2^>0.8) of CASP10 loci.


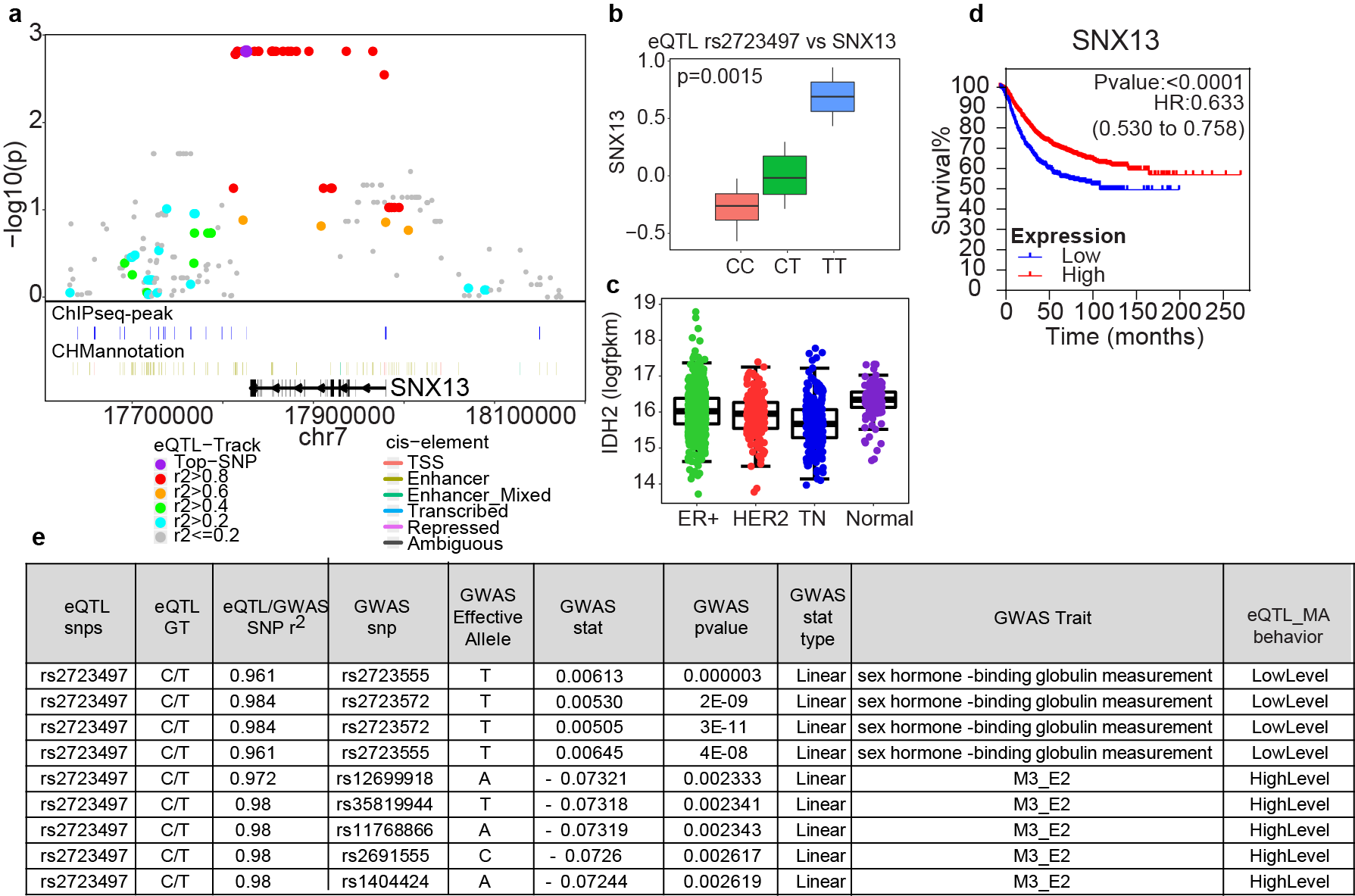


**Supplementary Figure S10: rs2723497-SNX13 Loci was implicated in multiple GWAS of sex hormone level. a** Locus zoom of rs2723497-SNX13 locus. PGx-eQTL was presented as –log_10_(p-value) against genomic coordinates. The top SNP is colored purple, and the rest of the SNPs are colored based on their genotyping correlation with the top SNP. AR ChIPseq peaks and ChromHMM annotation of all SNPs of the loci are labeled under respective SNP. **b** DHT induced log fold change (y-axis) of SNX13 expression by genotype of rs2723497. **c** Expression of IDH2 by breast cancer subtypes compared to non-tumoral normal tissue in TCGA breast cancer cohort. **d** Relapse free survival analysis of multiple cohorts of breast cancer, grouped by expression of SNX13. (D) List of PGx-eQTL SNPs and GWAS signals that is either exact matching or within LD (r^2^>0.8) of SNX13 loci.

**
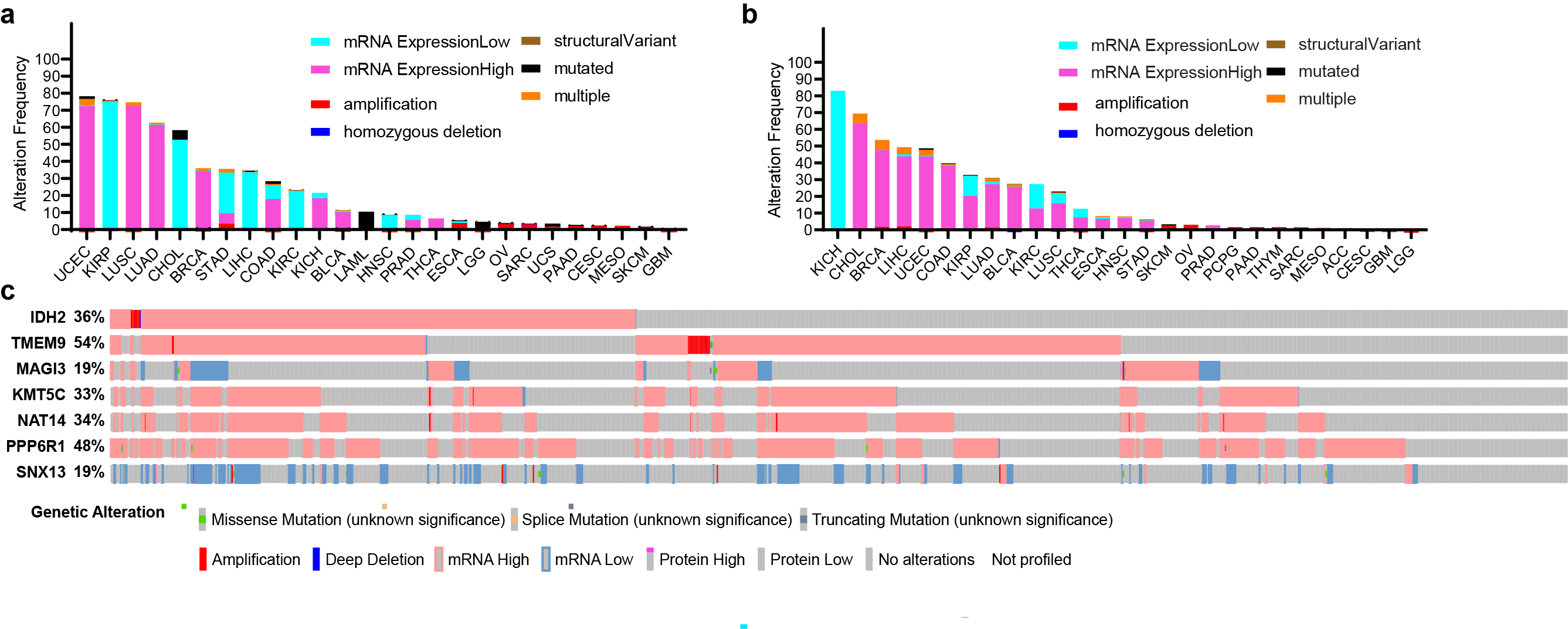
 Supplementary Figure S11: Somatic variants of selected PGx-eQTL and GWAS cross-identified genes from TCGA cohorts. a, b** Somatic variants of **a** IDH1 or IDH2, and **b** TMEM9 among all TCGA cohorts, generated by cBioportal. **c** Oncomap of selected PGx-eQTL genes among TCGA breast cancer cohorts, generated by cBioportal.
